# Supplementary material for: Irregular word reading as a marker of semantic decline in Alzheimer’s disease: implications for premorbid intellectual ability measurement
Source: Alzheimers Res Ther. 2024 May 2;16:96. doi: 10.1186/s13195-024-01438-3 (PMC11064305; doi:10.1186/s13195-024-01438-3)
Supplement: Supplementary file 1 — Additional file 1: Supplementary Table 1. Contrasts between diagnostic categories on AmNART total error scores. [file 13195_2024_1438_MOESM1_ESM.docx]

| **Supplementary Table 1** Contrasts between diagnostic categories on AmNART total error scores | | | |
| --- | --- | --- | --- |
| Contrast | Cohen's *d* | *t* | *P* |
| HC - SCD | -0.14 | -1.32 | .68 |
| HC - EMCI | -0.25 | -3.48 | < .01 |
| HC - LMCI | -0.34 | -5.97 | < .001 |
| HC - AD | -0.68 | -10.18 | < .001 |
| SCD - EMCI | -0.11 | -0.94 | .88 |
| SCD - LMCI | -0.20 | -1.89 | .33 |
| SCD - AD | -0.55 | -4.83 | < .001 |
| EMCI - LMCI | -0.09 | -1.31 | .69 |
| EMCI - AD | -0.44 | -5.44 | < .001 |
| LMCI - AD | -0.34 | -5.06 | < .001 |
